# Clinical observation and management of COVID-19 patients

**DOI:** 10.1080/22221751.2020.1741327

**Published:** 2020-03-25

**Authors:** Taisheng Li, Hongzhou Lu, Wenhong Zhang

**Affiliations:** a Department of Infectious Diseases, Peking Union Medical College Hospital, Peking Union Medical College, Chinese Academy of Medical Sciences, Beijing, People’s Republic of China; b Shanghai Public Health Clinical Center, Shanghai, People’s Republic of China; c Department of Infectious Diseases, Huashan Hospital, Shanghai Medical College, Fudan University, Shanghai, People’s Republic of China

**Keywords:** Clinical management, antiviral drugs, coronavirus, SARS, SARS-CoV-2

## Abstract

Three leading infectious disease experts in China were invited to share their bedside observations in the management of COVID-19 patients. Professor Taisheng Li was sent to Wuhan to provide frontline medical care. He depicts the clinical course of SARS-CoV-2 infection. Furthermore, he observes the significant abnormality of coagulation function and proposes that the early intravenous immunoglobulin and low molecular weight heparin anticoagulation therapy are very important. Professor Hongzhou Lu, a leader in China to try various anti-viral drugs, expresses concern on the quality of the ongoing clinical trials as most trials are small in scale and repetitive in nature, and emphasizes the importance of the quick publication of clinical trial results. Regarding the traditional Chinese medicine, Professor Lu suggests to develop a creative evaluation system because of the complicated chemical compositions. Professor Wenhong Zhang is responsible for Shanghai's overall clinical management of the COVID-19 cases. He introduces the team approach to manage COVID-19 patients. For severe or critically ill patients, in addition to the respiratory supportive treatment, timely multiorgan evaluation and treatment is very crucial. The medical decisions and interventions are carefully tailored to the unique characteristics of each patient.

Since late 2019 to early 2020, the sudden outbreak of COVID-19, among the general human population in the City of Wuhan, caused by the transmission of a novel coronavirus, SARS-CoV-2 [1,2], has led to over 80,000 diagnosed cases including more than 3000 deaths globally as of 29 February 2020. While the overall mortality rate for SARS-CoV-2 seems lower than SARS or MERS, the death rate among severe cases infected by SARS-CoV-2 is alarming. There are a wide range of efforts to develop special treatments for COVID-19 with limited success. In the current commentary, EMI invited three leading infectious disease experts in China who have personally participated in the clinical management of COVID-19 cases to share their bedside observations and to suggest what may be important in formulating the treatment strategy to optimize the clinical outcome.

## Professor Taisheng Li (being sent to Wuhan from Beijing to provide frontline care to the COVID-19 cases in a local hospital)

Since 7 February 2020, my colleagues and I went to work in the intensive care unit (ICU) of Zhongfa Xincheng Hospital, part of Tongji Hospital in Wuhan. Although the case fatality rate of COVID-19 is not high, due to the widespread of the disease, the total number of deaths is not small. Based on my observations in the treatment of severe and critically ill patients in the ICU, I propose that the early intravenous immunoglobulin (IVIG) and low molecular weight heparin (LMWH) anticoagulation therapy are very important.

### Clinical course of COVID-19

When SARS-CoV-2 infects a person, the lesions are not limited to the lungs. The virus causes viraemia after entering the body and the main clinical manifestations are fever, pharyngalgia, fatigue, diarrhoea and other non-specific symptoms [3,4]. This process includes the incubation phase and the early phase of the disease. The incubation takes 1–14 days (3–7 days being common). Peripheral blood leucocytes and lymphocytes are not significantly reduced (normal or slightly lower) at this phase. Then, the viruses spread through the bloodstream and mainly in the lungs, gastrointestinal tract, and heart, presumably concentrated in the tissues expressing ACE2, the receptor of SARS-CoV-2. This phase occurs around 7–14 after the onset of the symptoms when the virus starts a second attack, which is also the main cause of the aggravation of symptoms. At this time, pulmonary lesions became worse, and chest CT scans show imaging changes consistent with COVID-19. At this stage, the peripheral blood lymphocytes decrease significantly, involving both T and B lymphocytes. Inflammatory factors in peripheral blood are increased.

### Abnormal coagulation status

**Figure 1.**
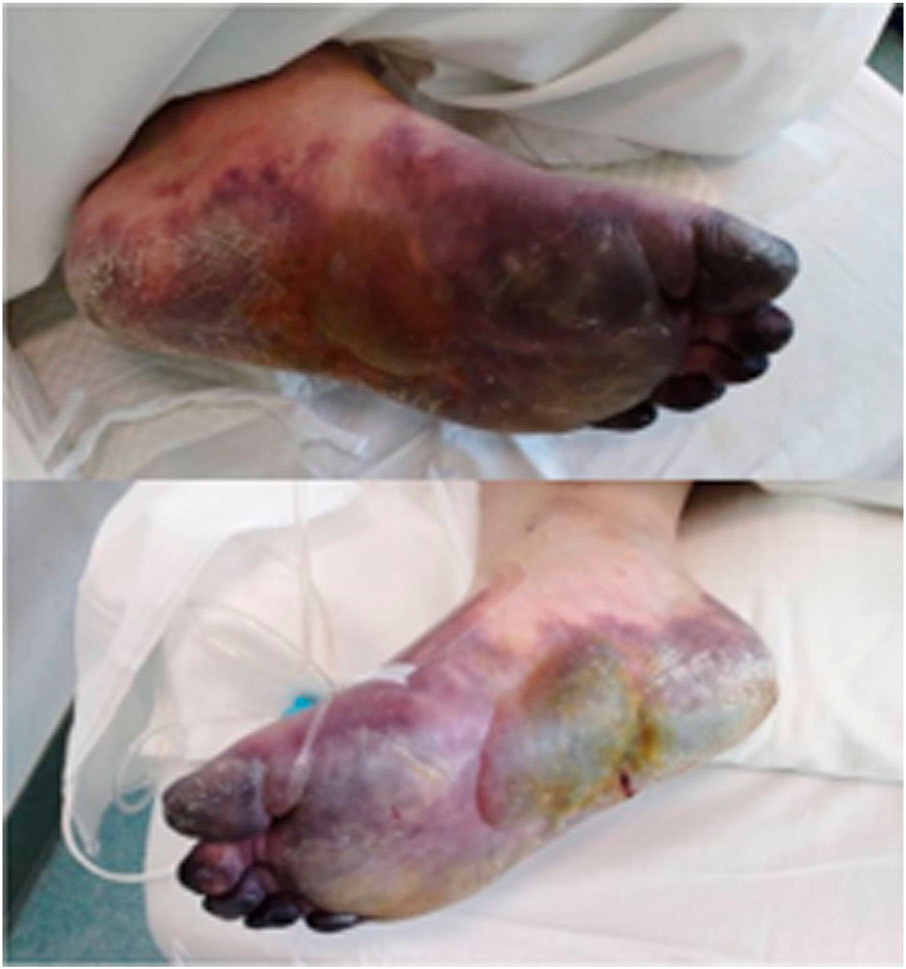
Ecchymosis in severe type of COVID-19 patients.

Patients at this phase will begin to develop the hypercoagulable state and D-Dimer-based coagulation factors may appear abnormal. The use of IVIG at this time may provide patients with effective clinical benefits and inhibit the formation of inflammatory factors storm (“cytokine storm”). LWMH therapy, suggested by colleagues at the Department of Hematology, Peking Union Medical College Hospital, may also alleviate the hypercoagulable state in patients. During the development of dyspnoea and chest imaging changes from light to severe, the D-dimer increased from mild to significant, along with prolonged prothrombin time (PT) and gradual decrease of fibrinogen (FBG) and platelet. Recently, it has been observed that some of the non-survivors suffered from ischaemic changes such as ecchymosis of the fingers and toes (Figure 1), at the same time as the organ functions of the heart and kidneys became worse. The above manifestations are consistent with the diagnosis of the hypercoagulable phase of DIC. It is believed that COVID-2019 can activate coagulation cascade through various mechanisms, leading to severe hypercoagulability. Early anticoagulation may block clotting formation and reduce microthrombus, thereby reducing the risk of major organ damages [5,6].

## Professor Hongzhou Lu (a leader in China to try various anti-viral drugs to treat COVID-19 cases)

Considering the clinical and epidemiological characteristics of COVID-19, the possible effective medications are in dire need to improve the prognosis of the patients and to stem the spread of the virus. More than 200 clinical trials have been organized mainly for the study of anti-viral drugs including Kaletra, Remdesivir, Arbidol, Favipiravir, Chloroquine, HCQ, Darunavir, Lianhua Qingwen Granules and Shuanghuanglian Oral Liquid. While the clinicians are enthusiastic about exploring the efficacy of these drugs, these trials are subject to all kinds of practical limitations. The majority of the trials are small in scale and repetitive in nature. For instance, a dozen clinical trials on Chloroquine have been carried out. Furthermore, due to the difference in design methodology, contradictory results may arise. The World Health Organization (WHO) has shown deep concern over the quality of these clinical trials and has provided guidance. A multitude of domestic experts also contributed their wisdom in different ways.

### Research design

What we are faced with is a newly discovered viral disease, testing drugs are selected mainly based on the past experience of such drugs treating other related diseases. Some of them only had in vitro tests. Some hasn’t finished Phase II clinical trials. Large-scale clinical trials will take a long time. Therefore, the small-scale clinical trials, including Phase II clinical trials, are currently given priority to, in which case, its effect, proper dosage, the medication and adverse reaction are initially verified. Those possibly effective and relatively safe medications can be coordinated to the large-scale and multi-centre clinical trials. In this way, a bulk of repetitive trials are avoided.

Another reality situation is the fact that the National COVID-19 Treatment Guideline has been updated quite urgently to include possibly effective drugs and experts are continuously offering new medical suggestions. Therefore, the medical workers in clinical trials usually take consideration of national guideline and expert opinion, while carrying out the trial under the premise that the normal treatment is not affected. As a result, the intervention group may be covered by a variety of anti-viral medications. It is difficult to leave the control group blank in order to confirm the absolute effect of the testing drug in the intervention group. The specific effects of the testing medication cannot be verified. In this case, the current clinical studies are not as rigorous as the traditional clinical trials, but they help draw a quick initial result which can help with the following study design. It is of vital importance to motivate the quick publication of clinical trial results, which can contribute significantly to clinical treatment.

### Traditional Chinese medicine

Traditional Chinese medicine, after a long history of clinical use, has carved out its own theory and practice. The most typical ones are the “Doctrine of Shanghai” (febrile diseases caused by exogenous pathogenic factors) and the “Doctrine of Seasonal Febrile Diseases.” In the diagnosis and treatment of acute respiratory infectious diseases like SARS and influenza, traditional Chinese medicine has played a significant role. But its treatment determination is based on “Syndrome Differentiation,” in which case, individual plans are produced. The Chinese medicine formulae are typical of complicated chemical compositions. This means that a creative evaluation system of traditional Chinese medicine needs to be developed.

## Professor Wenhong Zhang (responsible for Shanghai’s overall clinical management of the COVID-19 cases)

Since late January, I have been working to ensure the high-quality care of COVID-19 patient by building a highly experienced clinician team, Shanghai Clinical Treatment Expert Group, based on group members’ clinical and scientific expertise to provide the advice to complicated clinical management.

### A team approach to manage COVID-19 patients

Currently, there have been more than 330 laboratory-confirmed adult cases of COVID-19 in Shanghai [7]. Most patients (>90%) are in mild or moderate states, and more than 90% of them have been cured and discharged by now. Because no specific drugs have been shown with clear effectiveness in helping the human body to clear the SARS-CoV-2 viruses, the disease severity rate in Shanghai (26 cases, around 9–10%) actually objectively reflected the natural history of the disease, and was comparable with the severity rate at other places outside Hubei Province. All critically ill patients received invasive mechanical ventilation, and six patients received extracorporeal membrane oxygenation (ECMO). We found that in addition to the involvement of the respiratory system, critically ill patients often had systemic involvement of multiple organs, including the heart, kidney, and coagulation system in the early disease course. Sometimes multiple system involvement was observed even at the time of initial hospital admission. Therefore, in addition to the respiratory supportive treatment, timely multiorgan evaluation and treatment is very crucial.

Every critically ill COVID-19 patient in Shanghai is managed by a group of healthcare providers which includes at least a pulmonologist, an infectious diseases expert, a critical care specialist, and an ECMO specialist if necessary. Nephrologists, psychiatrists, physicians of traditional Chinese medicine, experienced nurses also actively participate in managing these patients as well. Besides, substantial heterogeneity among severe patients was noted. Mild patients are more alike, but each severe case is severe in his/her own way. There may be multiple pathophysiological mechanisms in these critically ill patients. The types of critically ill patients need to be more carefully classified in order to perform more individualized treatment, in addition to conducting more dedicated research to develop a unique management plan. In Shanghai, medical decisions and interventions are carefully tailored to the unique characteristics of each severe patient.

### Early and effective treatment of mild cases is critical

The overall mortality rate is about 0.9% in Shanghai. However, we have found that once the disease course progressed to the critical illness state (requiring mechanical ventilation), the prognosis of the patients would become significantly worse. From this perspective, treatments that can prevent mild state from progressing to the severe or critical state will significantly improve the overall prognosis of the clinical courses. Such effective treatments include intermittent short-term haemofiltration (ISVVH), low-dose short-course glucocorticoids therapy, among other approaches. The use of glucocorticoids is rather controversial and there is no general agreement by now. Based on our experiences, stably mild patients could self-manage the infection effectively and corticosteroid would not be recommended for them considering its potential risks. For patients who have an overly exuberant inflammatory response or are at high risk of developing ARDS, early-start of corticosteroids could be helpful. The benefit of corticosteroids as rescue treatment remains doubtful.

## References

[1] Chan JF, Kok KH, Zhu Z, et al. Genomic characterization of the 2019 novel human-pathogenic coronavirus isolated from a patient with atypical pneumonia after visiting Wuhan. Emerg Microb Infect. 2020;9:221–236. doi: 10.1080/22221751.2020.1719902 PMC7067204 31987001

[2] Chen L, Liu W, Zhang Q, et al. RNA based mNGS approach identifies a novel human coronavirus from two individual pneumonia cases in 2019 Wuhan outbreak. Emerg Microb Infect. 2020;9:313–319. doi: 10.1080/22221751.2020.1725399 PMC7033720 32020836

[3] Zhang W, Du RH, Li B, et al. Molecular and serological investigation of 2019-nCoV infected patients: implication of multiple shedding routes. Emerg Microb Infect. 2020;9:386–389. doi: 10.1080/22221751.2020.1729071 PMC7048229 32065057

[4] Chen WL, Lan Y, Yuan XZ, et al. Detectable 2019-nCoV viral RNA in blood is a strong indicator for the further clinical severity. Emerg Microb Infect. 2020;9:469–473. doi: 10.1080/22221751.2020.1732837 PMC7054964 32102625

[5] Working Group of 2019 Novel Coronavirus, Peking Union Medical College Hospital. Diagnosis and clinical management of Severe Acute Respiratory Syndrome Coronavirus 2 (SARS-CoV-2) infection: an operational recommendation of Peking Union Medical College Hospital (V2.0). Emerg Microb Infect. 2020;9:582–585. 10.1080/22221751.2020.1735265 PMC7103730 32172669

[6] Lin L, Lu LF, Cao W, et al. Hypothesis for potential pathogenesis of SARS-CoV-2 infection–a review of immune changes in patients with viral pneumonia. Emerg Microb Infect. 2020. 10.1080/22221751.2020.1746199 PMC7170333 32196410

[7] Ai J, Zhang Y, Zhang H, et al. Era of molecular diagnosis for pathogen identification of unexplained pneumonia, lessons to be learned. Emerg Microbes Infec. 9:1:597–600. 32174267 10.1080/22221751.2020.1738905 PMC7144283

